# Copolymerized Natural Fibre from the Mesocarp of *Orbignya phalerata* (Babassu Fruit) as an Irrigating-Fertilizer for Growing Cactus Pears

**DOI:** 10.3390/polym12081699

**Published:** 2020-07-29

**Authors:** Ricardo Edvan, Mariane Sá, Regina Magalhães, Rafael Ratke, Heldeney R. Sousa, Lucas Mateus Lima Neri, Edson C. Silva-Filho, Jose Pereira Filho, Leilson Bezerra

**Affiliations:** 1 Department of Animal Science, Federal University of Piauí, Bom Jesus, Piauí 64900000, Brazil; marivieira_15@hotmail.com (M.S.); regina_zootec@yahoo.com (R.M.); 2 Department of Agronomy, Federal University of Mato Grosso do Sul, Chapadão do Sul, Mato Grosso do Sul 79560000, Brazil; rafael.ratke@ufms.br; 3 Chemistry Department, Campus Ministro Petrônio Portela, Federal University of Piauí, Teresina, Piauí 64049550, Brazil; dine.17@hotmail.com (H.R.S.); luslima_neris@hotmail.com (L.M.L.N.); 4 Center of Health and Agricultural Technology, Federal University of Campina Grande, Patos, Paraiba 58708110, Brazil; jmorais@cstr.ufcg.edu.br

**Keywords:** cactus, fertilization, irrigation, polyacrylamide, polymers

## Abstract

Cactus pears face challenges due to global climate change, which is leading to in-depth research to monitor and increase their water activity. The objective of this study was to evaluate the use of the natural test hydrogel (TH) from *Orbignya phalerata* fibre as nutrients and water for growing cactus pear genotypes (“Baiana” and “Doce” [*Nopalea cochenillifera*], ‘Gigante’ [*Opuntia fícus-indica*], and “Mexican Elephant Ear” [*Opuntia stricta*]) compared to the use of commercial hydrogel (CH), which is based on polymers composed of polyacrylamide, and a treatment without the use of hydrogel (WH). A completely randomized design was used, in a factorial scheme (4 × 3), with four genotypes of cactus pear and three forms of hydration, with five replications. The number and area of cladode was greatest (*p* < 0.01) in plants with CH and TH irrigation-fertilization in the ‘Doce’ cactus genotype. The dry biomass of the cladode and root in the ‘Gigante’ cactus genotype was greatest (*p* < 0.01) in the treatments with CH and TH irrigation-fertilisation. The ‘Baiana’, ‘Doce’, and ‘Gigante’ cactus genotypes exhibited more (*p* < 0.01) dry matter content with the TH irrigation-fertilisation. The highest (*p* < 0.01) neutral detergent fibre content was observed in the ‘Baiana’ and ‘Doce’ cactus genotypes when irrigation occurred with WH treatment, and the highest acid detergent fibre content in the ‘Gigante’ genotype. The copolymerized natural fibre from the mesocarp of *Orbignya phalerata* (babassu fruit) induced a better growth and chemical composition of cactus pear genotypes than the hydrogel based on polymers composed of polyacrylamide.

## 1. Introduction

Polymers composed of polyacrylamide, called hydrogels, are available on the market [1] and are used as substrates to cultivate plants. This type of hydrogel is already widely used in planting and cultivation in agriculture and forestry [2], with the main objective of supplying water slowly to the roots of plants when water is scarce in the soil [3,4], thus meeting the water needs of plants in critical periods. According to Cock et al. [5], hydrogels developed with biodegradable polymers such as starch, chitosan, and modified natural fibers, cross-linked with potassium acrylate and acrylamide, are an interesting novelty for plant development. The use of natural plant fibers in the production of hydrogels will allow substitution of synthetic compounds such as polyacrylamide polymers, for ecologically more sustainable products with lower production costs [6,7].

Natural fibers are among the most abundant polymers in nature [8] and are easily processed and biodegradable [7], thus hydrogels with natural fibers are an alternative that can reduce the cost of production and is environmentally friendly. The *Orbignya phalerata* is a tropical palm tree in Brazil [9] that produces a fruit called babassu. About 200,000 tons per year of this nut is gown to produce 70,000 tons of oil. The babassu coconut has a fibrous, lignocellulolytic epicarp, comprising 15% of the dry weight of the fruit. The mesocarp is a light brown layer, located under the epicarp, which is to 20% of the dry weight of the fruit [10]. Through the copolymerization technique and other physical and chemical processes, such as graphitization, the natural fiber of the mesocarp of the *O. phalerata* fruit can be used in the production of hydrogel.

The hydrogel produced from *O. phalerata* fiber, in addition to providing water, may also provide plant fertilizers. Supplying of fertilizers through the hydrogel is an efficient alternative because water and nutrients are released slowly into the soil, thus preventing leaching [11].

The Food and Agriculture Organization of the United Nations [12] recognizes the potential of cactus pear and its importance for the development of arid and semi-arid regions, especially in developing countries, through the economic exploitation of various species, with sustainable consequences for the environment and food security. With the possibility of providing various products and by-products, for human and animal nutrition, human medicine, the cosmetics industry, and the production of natural additives, the cactus pear represents an alternative income for those who live in arid and semi-arid regions in different parts of the world [13]. Therefore, cactus pear cultivation has great economic importance for regions with a dry climate, for both the agricultural and livestock sectors [14,15,16]. Among the species of cactus pear most used in animal feed in dry regions are the species *Nopalea cochenillifera* and the species of the genus *Opuntia* [16]. 

Thus, use of a hydrogel in cactus pear irrigation-fertilization can provide more growth and ensure productivity, increasing food production in arid regions. Thus, we hypothesized that the use of the natural fiber of *O. phalerata* in the production of a hydrogel would provide greater growth and better chemical composition in cactus pear plants than the commercial hydrogel based on polymers composed of polyacrylamide. The objective of this study was to evaluate the use of the copolymerized natural fiber from the mesocarp of *Orbignya phalerata* (babassu fruit) as nutrients and water for the growth and chemical composition of four cactus pear genotypes, comparing with the commercial hydrogel from a polyacrylamide polymer compound, as well as control without any hydrogel (three irrigation-fertilization systems).

## 2. Materials and Methods 

### 2.1. Location and Experimental Design 

The experiment was carried out in a greenhouse located in the municipality of Bom Jesus, Piaui State, Brazil, with geographical coordinates 09°04′28″ South, 44°21′31″ West and an average altitude of 277 m. The region’s climate is classified as BSh according to the 1936 Köppen classification, with dry winters and rainy summers [17].

A completely randomized design was used, with five repetitions, in a factorial scheme (4 × 3), with four genotypes of cactus pear (“Baiana” and “Doce” [*Nopalea cochenillifera*], “Gigante” [*Opuntia fícus-indica*] and “Mexican Elephant Ear” [*Opuntia stricta*]) and three irrigation-fertilization systems [control without hydrogel (WH) and two types of soil irrigation-fertilization (commercial hydrogel—CH and test hydrogel—TH)]. Each experimental unit consisted of a plastic pot containing 9.0 kg of soil with a plant; a total of 60 pots were used.

### 2.2. Test Hydrogel Production and Physicochemical Characteristics of Test and Commercial Hydrogels 

To produce the TH, a sample of 2.0 g of babassu coconut mesocarp was dispersed in 30.0 mL of water, under agitation and nitrogen atmosphere to decrease the oxygen inhibiting effect in the radical polymerization reaction. Next, 0.024 g of potassium phosphate and 2.10 g of acrylamide monomer were added to the supernatant, then 0.016 g of KPS initiator, 0.024 g of *N′N′*-methylenebisacrylamide, and 100 mg of potassium bicarbonate. After 5 min of stirring and bubbling with nitrogen, 100.0 µL of the TEMED accelerator was added. The system was closed and kept under nitrogen and stirring until reaching the gel point. Thus obtained, the gel was washed in a 30% methanol/water solution to remove the acrylamide homopolymer, then it was dried by lyophilization, until a constant mass. The synthesized gels (1.0 g) were subjected to an alkaline hydrolysis reaction with 40.0 mL of NaOH (0.5 mol/L) to convert their amide groups into carboxylate, then they were washed and lyophilized. All reagents used were obtained from Aldrich (São Paulo, Brazil), with an analytical grade, without prior treatment. The mesocarp was kindly provided by the company BabCoall (Teresina, Brazil). 

For analysis and comparison between TH and CH (Table 1), visual analysis was performed to identify the colour, and water solubility was verified. Particle size was measured with a caliper, humidity determined by thermal analysis, on the SDT-600 equipment from TA-Instruments (New Castle, DE, USA) under nitrogen flow. The ionic character of the hydrogel was subjected to the test of different pH values (acidic and basic), obtaining a better behavior of the material in the basic medium (anionic carboxylate groups).

### 2.3. Characterizations

The lyophilized hydrogel of the mesocarp reticulated with acrylamide with fertilizer added was characterized by scanning electron microscopy (SEM) and thermal analysis (TG/DTG).

#### 2.3.1. Scanning Electron Microscopy (SEM)

The micrographs were performed using a scanning electron microscope (SEM) with a field emission gun, brand FEI, model Quanta FEG 250, with acceleration voltage from 1 to 30 kV, equipped with EDS of SDD (Silicon drift detectors), Ametek brand, model HX-1001, Apollo X-SDD detector. The analysis was carried out at the Interdisciplinary Laboratory of Advanced Materials (LIMAV) of the Federal University of Piaui. To perform the micrographs, the sample was fixed on an aluminum substrate (stub) using double-sided carbon adhesive tape and covered with Au in the metallizer, Quorum brand, model Q150R, for 30 s, at 20 mA, by plasma generated in argon atmosphere. 

#### 2.3.2. Thermogravimetric Analysis (TG and DTG) 

Thermogravimetric analysis: TG—thermogravimetry and DTG—derived thermogravimetry, were performed on TA Instruments SDT-Q600 Simultaneous (Interdisciplinary Laboratory for Advanced Materials-LIMAV), with the objective of evaluating thermal stability and variations in mass and energy as a function of temperature. The analysis was carried out under an argon atmosphere, with a flow of 100 mL/min and heating speed of 10 °C/min, in a temperature range of 25 to 800 °C, from the alumina sample support.

### 2.4. Hydrogel Irrigation-Fertilisation

This study compared the application of a test hydrogel (TH), added with nutrients. The TH was based on the mesocarp of the babassu palm tree (*O. phalerata*) and was produced through the co-polymerization process. Each gram of TH contained 0.5% K_3_PO_4_, in the proportion of 67% K and 33% P. The TH had a swelling capacity of 1100 g of H_2_O/g of hydrogel, and it was non-toxic. The conditions used for the swelling test were 27 ± 2 °C and the pH was 7.0 ± 0.2.

The commercial hydrogel (CH) used was a hydrogel already commercialized based on copolymer of acrylamide and potassium acrylate of the Hydroplan-EB (SAP)^®^ brand. Both irrigation-fertilization hydrogels were applied to the soil for planting, already hydrated with the equivalent to 400 L for each kg of hydrogel, following the recommendation of the commercial hydrogel. In treatments with the CH and WH, the equivalent of the minerals (K and P) existing in the TH was applied, using as a source of simple superphosphate (18% P_2_O_5_ and 20% Ca^2+^) and potassium chloride (60% K_2_O_5_). In the treatment with TH, the equivalent of calcium was applied with hydrated lime (46% Ca^2+^). These treatments were diluted in water to facilitate application, with the aid of a syringe, placed in the soil at the time of planting.

### 2.5. Experimental Units

The experimental units consisted of plastic pots (27.5 cm top width, 22.1 cm bottom width, 24.7 cm height/9.0 L capacity, Nutriplan^®^, São Paulo, Brazil) containing dystrophic red-yellow Latosol soil associated with quartz sand [18], with sandy physical characterization (clay: 220 g/kg; silt: 50 g/kg; and sand 720 g/kg), collected at a depth of 0 to 0.20 m in an area of native forest in Brazil close to the greenhouse. After collection, the soil was sieved in a 6.00 mm diameter metal mesh to remove impurities.

The correction of acidity and fertilization of the soil were based on the chemical analysis of the soil, which presented the following chemical characteristics: 4.00 pH in water; 3.46 mg/dm phosphorus (P); 25.30 mg/dm potassium (K); 0.16 cmolc/dm^3^ calcium (Ca); 0.05 cmolc/dm^3^ magnesium (Mg); <0.30 cmolc/dm^3^ aluminum (Al); 4.62 cmolc/dm^3^ hydrogen + aluminum (H + Al); 0.22 cmolc/dm^3^ sum of bases (SB); 4.84 cmolc/dm^3^ CTC at pH 7.0 (T); 32.6% base saturation (V); and 0.2% saturation by aluminum (M).

The soil acidity was corrected by applying a dose equivalent to 2 t/ha of limestone filler (PRNT 92%; Dimy^®^, São Paulo, Brazil), calculated to increase the base saturation to 45%. To correct potassium levels, potassium chloride (60% K_2_O) was used as a potassium source, applying the equivalent of 40 kg of K_2_O/ha. To correct phosphorus levels, simple superphosphate (18% P_2_O_5_ and 20% Ca^2+^) was applied at 90 Kg of P_2_O_5_/ha. In addition, the equivalent of 100 kg of N/ha nitrogen in the form of urea (45% N) was applied. All soil corrections and fertilizations followed recommendations by Martha Jr. et al. [19]. The fertilizers were diluted in water and applied to the soil. In addition, K and P correction was also performed, for treatments with commercial hydrogel and without hydrogel, as previously described.

**Figure 1:**
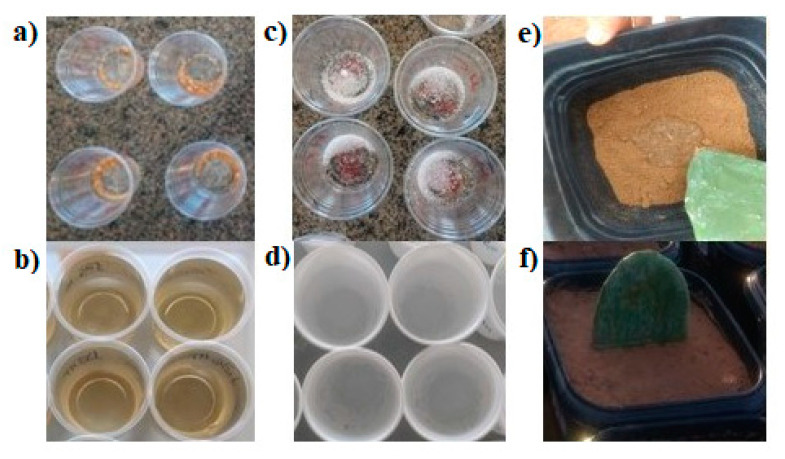
(**a**) Test hydrogel (TH); (**b**) TH hydrated; (**c**): commercial hydrogel (CH); (**d**) CH hydrated; (**e**) application of hydrogels to the soil before planting; (**f**) cactus pear planted with hydrogels.

The cactus pear cladodes used for planting in plastic pots were collected at the Federal University of Piaui. The cladodes had a healthy appearance and dark green color, were free from pests and diseases, and had similar ages and weights according to each genotype. The intermediate part of the plant cladodes was used, discarding the young and old cladodes. After selection, they were placed in a shaded place for healing, and ten days after the harvest, they were planted in the experimental units (pots). At the time of planting, the irrigation-fertilization hydrogels were applied according to the treatments; 0.4 g hydrogel/pot (equivalent to 20 kg per hectare) was applied, following the recommendation of the CH for sandy soils (72% sand in physical composition). The irrigation-fertilization hydrogels were placed in the bottom of each pot and already hydrated with water (Figure 1). 

To determine irrigation-fertilization, the pot capacity method, described by Casaroli and Lier [20], was applied. After determining the plastic pots capacity, irrigation was carried out through the daily weighing of the pots, measuring water stress on the plants, until a level of 10% of the field capacity was reached, with the aim to verify the potential of the hydrogels.

### 2.6. Analysis of Cactus Pear Growth 

The cactus pear genotypes growth was analyzed at out 180 days after planting. The following morphometric observations were made to characterize the growth of the plants: number of cladodes, obtained by counting the cladodes and the cactus pear height, measured with a tape measure (100 cm) from the soil surface to the apex of the highest cladode. 

The length (horizontal part) and width (vertical part) of the cladodes were measured in the central region of the cladodes with the aid of a measuring tape (100 cm), to obtain the cactus cladode area (cm^2^/plant) using the equation AC = length × width × 0.632, as described by Cortázar and Nobel [21]. To harvest the plant, a close-to-ground cut was made with the aid of a machete (Tramontina^®^, Carlos Barbosa, Brazil). 

The harvested material was weighed in the field, to obtain the total green biomass. Then it was placed in a forced ventilation oven at 65 °C, where it remained for 72 h, to obtain the dry weight according to the Association of Official Analytical Chemists (AOAC; method 967.03) [22]. Soon after, the dry samples were weighed to determine the dry biomass of cladodes (g/plant). In each pot, the roots were collected, washed with running water, and dried on paper towels to separate the soil contained in each pot. The samples were packed in paper bags and weighed, to obtain the fresh biomass. Then, they were taken to the forced ventilation oven to dry, at a temperature between 55 and 65 °C, until constant weight. Subsequently, the material was weighed on a precision scale to obtain the dry biomass of roots (g/plant).

### 2.7. Chemical Composition Analysis

Laboratory analyses were performed to determine the chemical composition (in triplicate) according to the recommendations of the AOAC [22]. Dry matter—DM (method 967.03), crude ash—CA (method 942.05), crude protein—CP (method 981.10), and ether extract—EE (method 920.29) were determined. The neutral detergent fiber (NDF) and acid detergent fiber (ADF) were determined according to the methodology proposed by Van Soest et al. [23], with the NDF residue incinerated in an oven at 600 °C for 4 h for the correction of ash and protein contamination. The samples were treated with thermostable alpha-amylase without the use of sodium sulfite. To determine acid detergent lignin (ADL), the ADF residue was treated with 72% sulfuric acid. 

### 2.8. Statistical Analysis

The experimental employed a randomized block design, arranged in a 4 × 3 factorial, with four cactus pear genotypes and three irrigation-fertilization systems. The following mathematical (Equation (1)) model was used:

*Yijk* = µ + *αi* + *βj* + (*αβ*)*ij* + *k* + *eijk* (1)

where *Yijk* is the observed value; µ is overall average of the treatment; *αi* is effect of cactus pear genotypes *i*; *i* is “Baiana” and “Doce” (*N. cochenillifera*), “Gigante” (*O. fícus-indica*), and “Mexican Elephant Ear” (*O. stricta*); *βj* is effect of irrigation-fertilization system j; j is control or WH and two other types of soil irrigation-fertilization (CH and TH); (*αβ*)*ij* is the effect of the interaction of the cactus pear genotypes and irrigation-fertilization system; and *eijk* is random error associated with each observation.

The studied variables were statistically treated by analysis of variance (ANOVA), followed by Scott-Knott test using software SISVAR^®^ version 5.0 [24]. All analyses were performed considering significant for *p*-value ≤ 0.05.

## 3. Results

**Figure 2:**
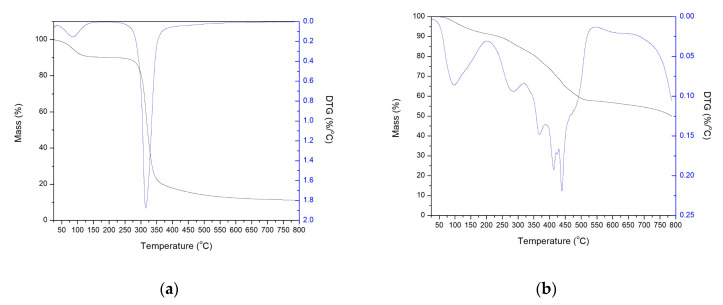
TG and DTG curves: (**a**) babassu mesocarp and (**b**) lyophilized hydrogel of the mesocarp reticulated with acrylamide with fertilizer added.

Figure 2 illustrates the results of the thermal analysis (TG and DTG) for the babassu mesocarp (a) and for the lyophilized hydrogel of the mesocarp reticulated with acrylamide with fertilizer added (b).

**Figure 3:**
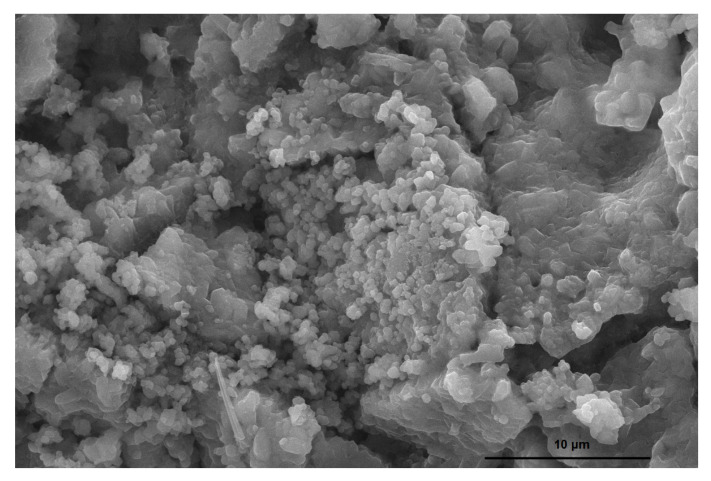
Scanning electron micrograph (SEM) of lyophilized hydrogel from babassu coconut mesocarp reticulated with acrylamide.

Figure 3 presents the scanning electron microscopy (SEM) of the lyophilized hydrogel of the mesocarp reticulated with acrylamide with fertilizer added.

There was an interaction (*p* < 0.01) between cactus pear genotypes and irrigation-fertilization sources for all plant growth characteristics (Table 2). A greater (*p* < 0.01) number of cladodes was achieved for the cactus pear irrigated-fertilized with the commercial hydrogel (CH) and test hydrogel (TH) with the “Doce” cactus genotype, with 5.5 ± 0.3 and 6.1 ± 0.3 cladodes per plant. The treatment without hydrogel (WH) produced fewer cladodes for all cactus pear genotypes. The “Gigante” cactus genotype grew more cladodes when cultivated with TH irrigation-fertilization. There was no difference (*p* > 0.01) between the irrigation-fertilization sources for the “Mexican Elephant Ear” genotype.

The “Doce” cactus pear genotype exhibited the largest (*p* < 0.01) area of the plant cladode with application of CH and TH irrigation-fertilization, obtaining 2060 ± 185 and 2479 ± 185 cm^2^/plant area, respectively. The treatment with TH obtained a greater area of plant cladode for the “Gigante” genotype. The “Mexican Elephant Ear” genotype showed no significant difference for the irrigation-fertilization source of the plant in relation to the area of the cladode.

The “Gigante” cactus genotype with WH irrigation-fertilization achieved a greatest (*p* < 0.01) plant height of 72.5 ± 2.2 cm. The “Doce” cactus genotype grew taller with the CH and TH treatments, at 45.0 ± 2.2 and 42.3 ± 2.2 cm, respectively. The “Mexican Elephant Ear” cactus genotype had greater height with the WH irrigation-fertilization treatment. However, the “Baiana” cactus genotype exhibited no effect (*p* > 0.01) in relation to the irrigation-fertilization of the plant. 

For dry biomass of cladodes, the “Gigante” cactus genotype obtained a greater (*p* < 0.01) amount with the CH and TH irrigation-fertilization, at 547 ± 15 and 546 ± 15 g/plant. The “Baiana” and “Doce” cactus genotypes presented more dry biomass of cladodes with the hydrogel treatments (CH and TH irrigation-fertilization). For the “Mexican Elephant Ear” genotype, there was no effect (*p* > 0.01) on dry biomass of cladodes with the different forms plant hydration. The highest values (*p* < 0.01) of dry biomass of roots were observed in the “Gigante” cactus genotype with the CH and TH irrigation-fertilization, at 11.3 ± 0.9 and 9.6 ± 0.9 g/plant, as well as in the “Mexican Elephant Ear” genotype with the TH irrigation-fertilization, at 11.4 ± 0.9 g/plant. In the “Doce” genotype, more dry biomass of roots was found with hydrogel treatments (CH and TH irrigation-fertilization), and in the “Baiana” cactus genotype, it was greatest with the CH treatment.

The evaluation of the chemical composition (Table 3) observed an interaction (*p* < 0.01) between the cactus pear genotypes and irrigation-fertilization sources of the plant for dry matter (DM), neutral detergent fibre (NDF), and acid detergent fiber (ADF). The contents of crude ash (CA) and organic matter (OM) were no effect (*p* > 0.05) for the different genotypes and hydration of the plant. However, for the crude protein (CP) content, there was an isolated effect (*p* < 0.01) for the different cactus pear genotypes and irrigation-fertilization sources.

The “Baiana”, “Doce”, and “Gigante” cactus genotypes presented a dry matter content (*p* < 0.01) with the TH irrigation-fertilization of 65, 69, and 63 g/kg DM, respectively. The “Mexican Elephant Ear” genotype achieved a higher DM content with CH irrigation-fertilization, obtaining 69 g/kg when fed (Table 3). The “Baiana” and “Doce” cactus genotypes presented the highest NDF content with the WH irrigation-fertilization treatment, obtaining 296 and 324 g/kg in DM basis, respectively. The greatest ADF content was observed for the “Gigante” cactus genotype with the WH irrigation-fertilization (122 g/kg in DM). The “Mexican Elephant Ear” genotype had greater CP content (123 g/kg in DM basis) than the other cactus pears genotypes, and the WH irrigation-fertilization obtained a higher CP concentration (113 g/kg in DM basis) than CH and TH.

## 4. Discussion

The thermogravimetric curve and the thermogravimetric derivative (Figure 2a) of the babassu coconut mesocarp indicates that the thermal decomposition of this material occurs in two stages, the first in the range of 35 to 150 °C, with a 10% weight loss, which is related to the dehydration of the material (vaporization of physically adsorbed water) [25,26].

The TG/DTG curves illustrate that two main thermal events occurred, the first in the range of 50 to 157 °C, with a mass loss of 6.4%, and a maximum rate of loss in temperature of 96 °C, being associated with vaporization of physically adsorbed water.

The second thermal event occurred together with a series of secondary reactions (and decompositions) and extends to 544 °C, with a mass loss of 33.1%, and a maximum decomposition temperature of 439 °C. This event is related to the initial degradation of the material, starting the depolymerization process, initially by breaking and volatilization of more superficial groups, followed by breaking and decomposition of larger groups [27], with little variation in mass after this temperature. The residual mass comes from the presence of the fertilizer.

The micrograph of the lyophilized hydrogel of the mesocarp reticulated with acrylamide with fertilizer added is presented in Figure 3. After the crosslinking a change occurred, as the babassu mesocarp has a spherical shape, very similar to starch microscopy [28] and after modification, a heterogeneity in the size of the particles was caused by the crosslinking, followed by the absorption and lyophilization of the water. A similar result was observed in thermogravimetry with several mass events in very close regions, caused by different particle sizes, altering its stability, which is ideal for application in systems to control release of water and fertilizers on the soil. Its microstructure contains pores distributed by its extension, which greatly help in the process of water absorption by the material, with very varied porosities, caused by the size of the particles, generating an excellent benefit for the controlled release [29]. The smallest particles found in the image are the fertilizer added to the system.

The interaction between the cactus pear genotypes and the different forms to hydrate the plant demonstrates that the morphophysiological differences of the plants and the use of the irrigation-fertilization hydrogel, as well as the type of hydrogel, influenced the main growth characteristics of the cactus pear. The evaluated cactus pear genotypes have very different morphological characteristics [30]. Hydrogel cultivation achieves greater hydration of the plants and, with this, greater growth [31].

The main characteristic of the cactus pear “Doce” genotype is the greater number of cladodes compared to the other genotypes evaluated [32]. More cladodes were observed with the use of hydrogels (CH and TH irrigation-fertilization), demonstrating the effect of these on cactus pear growth. An irrigation-fertilization hydrogel provides slow release of water and nutrients to the plant [33], stimulating growth of more cladodes when compared to WH irrigation-fertilization.

The TH produced with copolymerized natural fiber from the mesocarp of *Orbignya phalerata* (babassu fruit) exhibited potential, yielding a similar number of cladodes as CH irrigation-fertilization. Thus, natural fibers of *O. phalerata* have potential for production of hydrogels. According to Rodrigues et al. [34], natural fibers are abundant in nature, and their use is ecologically correct, in addition they can make low-cost products.

Both hydrogels produced a greater cladode area in cactus pear plants, which is mainly due to the water support provided by the hydrogel to the plants, since the plants were under water stress during the experimental period. The “Gigante” cactus genotype presented a greater number and area of cladodes with the use of the TH, demonstrating the irrigation-fertilization potential of the hydrogel produced from natural fibres of *O. phalerata*. The best performance for this genotype with the use of TH irrigation-fertilization occurred due to the slow release of water to the plant, associated with nutrients K and P [35]. With the CH irrigation-fertilization, the amount of K and P related to the compound in TH was applied to the soil. Using slow-release fertilizers avoids the leaching and volatilisation processes of nutrients in the soil [36].

The “Gigante” cactus genotype had greater plant height with the WH irrigation-fertilization, due to the morphological characteristic of this plant, which is taller than the other specimens evaluated. Thus, the greater plant height with the WH treatment can been explained due to the lower number and area of cladodes obtained with this treatment. Cactus pears with greater cladode development and more cladodes tend to have a lower plant height [37,38], which was what happened with the hydrogel treatments.

For the production of dry biomass of cactus pear cladodes, the “Gigante” cactus genotype obtained the highest biomass production due its large cladode size [39]. The “Baiana”, “Doce” and “Gigante” cactus genotypes showed higher dry biomass of cladodes with treatments using hydrogel (CH and TH irrigation-fertilization). The use of natural *O. phalerata* fibers and commercial hydrogels for the cactus pears increased growth characteristics because they improved the slow availability of water and fertilizers for plants over the WH treatment.

The irrigation-fertilization source did not change the number, area, and dry biomass of cladodes in the “Mexican Elephant Ear” cactus pear genotype. This genotype did not grow efficiently from the hydrogel application compared to the other genotypes of cacti studied, which may be related to the fact that the “Mexican Elephant Ear” genotype using water more efficiently; therefore, it did not respond to hydrogels irrigation [40,41].

The use of the irrigation-fertilization hydrogel also generated a greater amount of dry biomass of roots in the “Doce” and “Gigante” cactus genotypes, which presented better performance with the use of irrigation-fertilization hydrogels in the soil. The highest concentration of the root system of a cactus pear is in the superficial layer of the soil [42]. Cactus pears with a larger root system have greater production potential and withstand weathering conditions [43] as they have a greater capacity to absorb water and available nutrients in the soil [44].

The irrigation-fertilization hydrogel from copolymerized natural fiber from the mesocarp of *Orbignya phalerata* (babassu fruit) showed equal or better performance for the main growth characteristics of the evaluated cactus pears. The use of this type of natural fiber for hydrogel production has potential due to its availability [45] and economic and environmental advantage [46].

The greater dry matter (DM) content observed in the “Baiana”, “Doce”, and “Gigante” cactus genotypes with the use of TH irrigation-fertilization demonstrates that, this treatment facilitated greater accumulation of organic and mineral compounds in the tissues of the plants [33]. The greater DM content of the “Baiana”, “Doce”, and “Gigante” cactus genotypes is related to a higher total dry biomass accumulation, which is an important characteristic for animal production as it provides greater food availability, increasing food security for herds in dry regions [47].

Fiber is present in the cell wall of vegetables, formed primarily by cellulose, hemicellulose, lignin, protein, and other compounds. It has a priority function in ruminant nutrition, as a source of energy and to stimulate the fermentation processes [48,49,50]. The fiber digestion of forage is not constant for all animals or for all feeding conditions, varying from 135 to 780 g/kg in DM basis, but the main variation is due to differences in the structure, chemical composition, and maturity. Mertens [37] and the National Research Council (NRC) [51] suggested that a diet should have an added crude fiber source in the proportion of 5 to 20% of DM and between 250 to 330 g/kg of NDF and 100 to 150 g/kg ADF to stimulate the maximum intake of DM and ruminal health. The CH and TH irrigation-fertilization reduced ADF and increased NDF. According to Carvalho et al. [52], the cactus pear has a low fiber content, which can reduce the rumen pH and cause degradation of microbial flora if used as an exclusive ingredient in the diet of animals. An increase of fiber content in the plant indicates an increase in the cell wall, which improves the ruminal fermentation and feed digestibility for the ruminants [50,53]. 

The higher crude protein (CP) content obtained in the WH irrigation-fertilization in the cultivation of cactus pear was because the plant in this treatment suffered water stress, which in forage plants can increase their cellular content, especially CP content [54,55]. The application of fertilizers via localized irrigation is recommended, as small doses of micronutrients in a small volume of soil because conventional dosage can be phytotoxic. In the present study, the reduction in protein concentration was very small, which may be corrected when formulating animal diets. 

## 5. Conclusions

The use of the commercial hydrogel polymers from polyacrylamide compounds and test hydrogel from natural *O. phalerata* fiber improved growth and chemical composition in the cactus pear plants in pots, particularly of the “Doce” and “Gigante” genotypes. 

However, the irrigation-fertilisation with test hydrogel from copolymerized natural fiber from the mesocarp of *Orbignya phalerata* (babassu fruit) and presents similar production potential for cactus pear cultivation, compared to commercial hydrogel polymers from polyacrylamide compounds. Moreover, it is eco-friendlier with environmental and economic advantages due to the use of natural fibres. With the use of test hydrogel from natural *O. phalerata* fibre, water can be saved and thus contribute to the environment.

The test hydrogel from natural *O. phalerata* fibre has potential for hydration and fertilization of cactus pear plants and it should be tested as irrigation-fertilization in other plant cultures.

Field studies with a longer duration and repetition in time should be conducted with these cactus pear species, to obtain a more accurate recommendation for the use of this test hydrogel.

## Figures and Tables

**Table 1:** Physicochemical characteristics of hydrogels.

Characteristics	Commercial Hydrogel (CH) ^1^	Test Hydrogel (TH) ^2^
Ingredient	Polyacrylamide super absorbent	Mesocarp of babassu coconut reticulated with acrylamide
Particle size	0.5–3 mm	5–10 mm
Ionicity	Anionic	Anionic
Moisture	10%	6.4%
Density	0.8 g/cm	-/-
Colour	White	Brown
Form	Granulated solid	Granulated solid
Solubility in water	Insoluble	Insoluble

^1^ Commercial name Hydroplan-EB/HyA; Polymers composed of polyacrylamide superabsorbent. ^2^ Copolymerized natural fiber from the mesocarp of *Orbignya phalerata* (babassu fruit).

**Table 2:** Growth characteristic of four cactus pear genotypes under different forms of hydrogel irrigation-fertilization.

Genotypes (G)	Hydrogel Irrigation-Fertilization (HI)			Mean	Effect (*p*-Value) ^1^			SEM ^2^
	Without	Commercial	Test ^3^		HI	Genotype	HI × G	
Number of cladode (unit)								
‘Baiana’	2.1bA	3.3aB	3.5aC	2.6	<0.01	<0.01	<0.01	0.3
‘Doce’	2.4bA	5.5aA	6.1aA	4.5				
‘Gigante’	2.2cA	3.0bB	4.5aB	3.1				
‘MEE’ ^4^	2.0aA	1.5aC	2.0aC	1.8				
Mean	2.0	3.3	3.7					
Cactus cladode area (cm^2^/plant)								
‘Baiana’	551bA	1255aB	1020aB	746	<0.01	<0.01	<0.01	185
‘Doce’	314bA	2060aA	2479aA	1617				
‘Gigante’	796cA	1206bB	1965aA	1322				
‘MEE’	561aA	478aC	523aC	439				
Mean	555	1225	1313					
Cactus pear height (cm)								
‘Baiana’	40.0aC	38.9aC	40.7aB	39.8	<0.01	<0.01	<0.01	2.2
‘Doce’	37.0bC	45.0aB	42.3abB	41.4				
‘Gigante’	72.5aA	64.5bA	53.0cA	63.3				
‘MEE’	56.0aB	36.5bC	36.6bB	43.7				
Mean	51.3	46.2	43.6					
Dry biomass of cladode (g/plant)								
‘Baiana’	231bC	319aB	364aB	271	<0.01	<0.01	<0.01	15
‘Doce’	194bD	340aB	346aB	292				
‘Gigante’	434bA	547aA	546aA	542				
‘MEE’	262aB	246aC	240aC	293				
Mean	346	363	341					
Dry biomass of root (g/plant)								
‘Baiana’	4.0cA	10.3aAB	6.8bB	6.3	<0.01	<0.01	<0.01	0.9
‘Doce’	3.0bA	7.0aB	8.3aAB	6.1				
‘Gigante’	5.0bA	11.3aA	9.6aA	8.2				
‘MEE’	4.0bA	9.7aAB	11.4aA	8.3				
Mean	4.0	9.5	8.2					

^1^ Means followed by different lowercase letters in the row and uppercase letters in the column differ statistically by the Scott-Knott test *p* < 0.05. ^2^ SEM: standard error mean. ^3^ TH: Hydrogel produced from copolymerized natural fiber from the mesocarp of *Orbignya phalerata* (babassu fruit). ^4^ MEE: “Mexican Elephant Ear”.

**Table 3:** Chemical composition of four cactus pear genotypes under different irrigation-fertilization sources.

Genotypes (G)	Hydrogel Irrigation-Fertilization (HI)			Mean	Effect (*p*-Value) ^1^			SEM ^2^
	Without	Commercial	Test ^3^		HI	Genotype	HI × G	
Dry matter-DM (g/kg as fed)								
‘Baiana’	57Bb	66Aa	65Aa	63	<0.01	<0.01	<0.01	1.0
‘Doce’	63Ab	69Aa	69Aa	67				
‘Gigante’	60Aa	56Bb	63Aa	60				
‘MEE’ ^4^	56Bb	69Aa	56Bb	60				
Mean	59	65	63					
Crude ash-CA (g/kg dry matter)								
‘Baiana’	195	183	177	154	0.55	0.45	0.44	4.5
‘Doce’	174	178	178	177				
‘Gigante’	173	173	181	176				
‘MEE’ ^4^	166	162	190	173				
Mean	177	174	182					
Organic matter-OM (g/kg dry matter)								
‘Baiana’	804	816	822	814	0.55	0.45	0.44	4.5
‘Doce’	825	821	821	822				
‘Gigante’	826	826	818	823				
‘MEE’ ^4^	833	837	809	826				
Mean	822	825	817					
Crude protein-CP (g/kg dry matter)								
‘Baiana’	117	96	97	103B	<0.01	<0.01	0.10	2.1
‘Doce’	94	89	89	90C				
‘Gigante’	105	97	95	99B				
‘MEE’ ^4^	135	112	121	123A				
Mean	113a	98b	101b					
Neutral detergent fiber-NDF (g/kg dry matter)								
‘Baiana’	296Aa	218Bb	268Ba	261	<0.01	0.04	<0.01	7.1
‘Doce’	324Aa	227Bb	240Bb	264				
‘Gigante’	242Bb	242Bb	287Aa	257				
‘MEE’ ^4^	255Bb	281Ab	316Aa	284				
Mean	279	242	278					
Acid detergent fiber-ADF (g/kg dry matter)								
‘Baiana’	95Ba	80Bb	101Aa	92	<0.01	<0.01	<0.01	7.1
‘Doce’	95Ba	90Ba	102Aa	96				
‘Gigante’	122Aa	101Ab	97Ab	107				
‘MEE’ ^4^	89Ba	86Ba	91Aa	89				
Mean	100	89	98					

^1^ Means followed by different lowercase letters in the row and uppercase letters in the column differ statistically by the Scott-Knott test *p* < 0.05. ^2^ SEM: standard error mean. ^3^ TH: Hydrogel produced from copolymerized natural fiber from the mesocarp of *Orbignya phalerata* (babassu fruit). ^4^ MEE: ‘Mexican Elephant Ear’.
